# Safety, Pharmacokinetics, and Food Effect of the RORα Agonist TB-840, a Novel Candidate for Metabolic Dysfunction-Associated Steatohepatitis (MASH): A Randomized First-in-Human Study in Healthy Volunteers

**DOI:** 10.3390/life15091410

**Published:** 2025-09-07

**Authors:** Inyoung Hwang, Shi-Ra Lee, Heung Jae Kim, Yun Kim, Sang Won Lee

**Affiliations:** 1 Department of Clinical Pharmacology and Therapeutics, Hanyang University Seoul Hospital, Seoul 04763, Republic of Korea; isoleucine@snu.ac.kr (I.H.); jokomi2001@gmail.com (S.-R.L.); 2 Department of Pharmacology, Hanyang University College of Medicine, Seoul 04763, Republic of Korea; 3 Therasid Bioscience Inc., Seongnam-si 13488, Republic of Korea; jaykim@therasidbio.com; 4 College of Pharmacy, Daegu Catholic University, Gyeongsan 38430, Republic of Korea

**Keywords:** RORα agonist, metabolic dysfunction-associated steatohepatitis (MASH), metabolic dysfunction-associated steatotic liver disease (MASLD), first-in-human, pharmacokinetics, food effect

## Abstract

TB-840 is a novel RORα agonist being developed by Therasid Bioscience Inc. for the treatment of metabolic dysfunction-associated steatohepatitis (MASH). This first-in-human study evaluated the safety, tolerability, pharmacokinetics, and food effect of single ascending doses of TB-840 in healthy adult volunteers. In the single ascending dose part, 64 participants were randomized to receive TB-840 (12.5–200 mg) or placebo. In the food effect part, 6 participants received a single 200 mg dose under fasted and fed conditions in a crossover design. TB-840 was rapidly absorbed (median T_max_ 1.7–2.5 h) with a mean half-life of 4.8–9.7 h. Systemic exposure increased dose-proportionally across the studied dose range. A high-fat meal delayed absorption and increased the systemic exposure. TB-840 was well-tolerated, with no serious adverse events reported. These results support the continued development of TB-840 as a potential treatment for MASH. Further studies are warranted to evaluate its efficacy and safety in the target patient population (ClinicalTrials.gov identifier: NCT05045534).

## 1. Introduction

Metabolic dysfunction-associated steatohepatitis (MASH), formerly known as nonalcoholic steatohepatitis (NASH), is a severe and progressive form of metabolic dysfunction-associated steatotic liver disease (MASLD), formerly known as nonalcoholic fatty liver disease (NAFLD). MASLD has become a global epidemic, affecting approximately 30% of the adult population [1]. MASLD is defined as the accumulation of lipids in over 5% of hepatocytes and can progress to MASH, which is characterized by concomitant hepatic inflammation and hepatocyte injury [2]. In turn, MASH can progress to hepatic fibrosis and eventually to cirrhosis, which carries a 2–3% annual risk of developing into hepatocellular carcinoma [3]. Despite this significant clinical burden, the therapeutic landscape for MASH has long been characterized by profound unmet medical needs, with lifestyle interventions, including diet and exercise, representing the only recommended management strategies for many years [4].

The pathophysiological complexity and heterogeneity of MASH pose significant challenges in drug development, contributing to numerous late-stage clinical trial failures [5]. Other major hurdles include high and variable placebo response rates and the reliance on invasive liver biopsies as the primary regulatory endpoint [6,7,8]. However, the approval of resmetirom (Rezdiffra^®^, Madrigal Pharmaceuticals, Inc., West Conshohocken, PA, USA) in March 2024 by the U.S. Food and Drug Administration (FDA) marked a significant milestone [9]. As the first pharmacotherapy approved for adults with noncirrhotic MASH and moderate to advanced liver fibrosis, resmetirom has validated the thyroid hormone receptor β (THRβ) pathway as a key therapeutic target [10]. This landmark approval has helped to establish a clearer regulatory pathway for the development of other MASH therapies.

Building on this progress, a robust pipeline of therapeutic agents is essential for addressing the multifaceted nature of MASH. Developmental agents can be broadly categorized according to their primary mechanisms of action. Some primarily target systemic metabolic dysregulation driven by chronic overnutrition, including incretin analogs, such as glucagon-like peptide-1 (GLP-1) receptor agonists (e.g., semaglutide) and fibroblast growth factor-21 (FGF-21) receptor agonists (e.g., efruxifermin) [11,12]. In contrast, other drug classes directly target hepatic pathways, including nuclear receptor ligands such as peroxisome proliferator-activated receptor (PPAR) agonists (e.g., lanifibranor), and de novo lipogenesis inhibitors such as acetyl-CoA carboxylase (ACC) inhibitors (e.g., firsocostat) and fatty acid synthase (FAS) inhibitors (e.g., TVB-2640) [13,14,15]. Furthermore, combination therapies that target multiple pathogenic pathways are a key area of investigation, as this approach may enhance the efficacy and improve the safety profile of individual agents. This strategy has highlighted the need for novel drugs with diverse mechanisms of action.

Retinoid-related orphan receptor alpha (RORα), a member of the NR1 subfamily of nuclear receptors, participates in various biological processes, including the circadian rhythm, lipid metabolism, and inflammation [16,17]. RORα has recently emerged as a compelling therapeutic target for MASH, as its expression is reportedly lower in patients with MASLD [18]. In murine models, RORα activation has been found to protect against the development of MASH under nutrient overload conditions by attenuating hepatic steatosis, oxidative stress, and inflammation, while enhancing mitochondrial quality control and promoting the polarization of liver macrophages toward an anti-inflammatory M2 phenotype [19,20,21,22,23]. Furthermore, RORα was recently demonstrated to contribute to the maintenance of genomic integrity by preventing NAFLD-induced polyploidization [24].

TB-840 (formerly JC1-40) is a novel, synthetic, and orally available thiourea derivative that acts as an RORα agonist and is under development by Therasid Bioscience Inc. (Seongnam-si, Republic of Korea) [25]. In a high-fat diet (HFD)-induced fatty liver mouse model, the administration of TB-840 was found to lower hepatic triglyceride levels and ameliorate steatosis [19]. Furthermore, in a methionine/choline-deficient (MCD) diet-fed NASH mouse model, TB-840 decreased liver injury, lipid peroxidation, and inflammation [20]. TB-840 also improved NASH symptoms in an HFD-induced mouse model by promoting M2 polarization of liver macrophages [22]. These promising preclinical findings provide a rationale for advancing TB-840 into clinical development. This first-in-human phase 1 study aimed to evaluate the safety, tolerability, and pharmacokinetic (PK) profile of single ascending doses (SADs) of TB-840 and to assess the effect of food on its bioavailability in healthy adult volunteers.

## 2. Materials and Methods

### 2.1. Study Design

This randomized, double-blind, placebo-controlled, dose-escalation phase I study was conducted at Hanyang University Seoul Hospital in Seoul, Republic of Korea. The study comprised an SAD component and a food effect (FE) component. The dose levels evaluated in the SAD component were 12.5, 25, 37.5, 50, 75, 100, 150, and 200 mg, whereas a 200 mg dose was used for the FE component.

The starting dose was determined from nonclinical toxicology studies in accordance with FDA guidelines [26]. A no-observed-adverse-effect level (NOAEL) of 15 mg/kg has been established in dogs, which have been identified as the most sensitive species, yielding the lowest human-equivalent dose (HED) of 8.1 mg/kg. A conservative safety factor of 30 was applied to this HED to maximize participant safety, as this trial is the first to evaluate an RORα agonist in humans. This calculation resulted in a maximum recommended starting dose (MRSD) of 16.2 mg for a 60 kg adult; consequently, the initial dose was set at 12.5 mg based on the available dosage form. The maximum planned dose of 200 mg was selected because it was twice the predicted effective dose of 100 mg derived from a physiologically based pharmacokinetic (PBPK) model. This dose range was chosen to assess the dose proportionality within a potential therapeutic range and to inform the design of subsequent studies.

In each SAD group, 8 participants were randomized using a computer-generated schedule in a 6:2 ratio to receive a single oral dose of TB-840 or a matching placebo. All the participants, investigators, site personnel, and sponsor staff involved in this study remained blinded to the treatment allocation until the database was locked. Although not based on formal statistical power calculations, the cohort sizes were consistent with the standard practice for exploratory first-in-human studies and were deemed sufficient for the initial characterization of the safety, tolerability, and pharmacokinetic profile. The study drug was administered in 200 mL of water after an overnight fast for at least 10 h. Dose escalation to the next level proceeded only after the investigators and sponsor completed a thorough review of the safety and tolerability data from the preceding cohort.

The FE part of this study was conducted as an open-label, two-period, fixed-sequence crossover study with a single cohort of six participants. Each participant received a 200 mg oral dose of TB-840 under fasting conditions (Period 1). After a washout period of at least 7 days, they received the same dose in Period 2, 30 min after starting a standardized high-fat, high-calorie breakfast (approximately 900 kcal, with at least 40% of the calories from fat), in accordance with regulatory guidelines to assess the maximal potential effect of food on the bioavailability of TB-840.

The study was performed in compliance with the ethical principles of the Declaration of Helsinki, International Council for Harmonisation (ICH) Good Clinical Practice (GCP) guidelines, and all applicable local regulatory requirements. The study protocol and informed consent forms were reviewed and approved by the Korean Ministry of Food and Drug Safety (MFDS) and the Institutional Review Board (IRB) of Hanyang University Seoul Hospital (approval number: HYUH-2021-04-061). All participants provided written informed consent after receiving a comprehensive explanation of the study procedure. The trial was registered at ClinicalTrials.gov (Identifier: NCT05045534; date of registration: 14 September 2021).

### 2.2. Study Participants

Eligible participants were healthy Korean adult males between the ages of 19 and 45 years with a body mass index (BMI) ranging from 18.0 to 27.0 kg/m^2^. This specific population was selected to minimize potential sources of pharmacokinetic variability, such as hormonal fluctuations related to the menstrual cycle and genetic polymorphisms in drug-metabolizing enzymes that can differ between ethnic groups. Eligibility was assessed on the basis of medical history, physical examination, vital signs, 12-lead electrocardiography (ECG), clinical laboratory tests, serology, and urinary drug screening. The major exclusion criteria were any clinically significant history or the presence of hepatic, renal, gastrointestinal, cardiovascular, endocrine, or neuropsychiatric diseases. Clinical laboratory test-based exclusion criteria included aspartate aminotransferase (AST), alanine aminotransferase (ALT), alkaline phosphatase (ALP), total bilirubin, gamma-glutamyl transferase (GGT), and serum creatinine levels > 1.5 × the upper limit of normal (ULN).

### 2.3. Determination of Plasma and Urine Concentrations of TB-840

The plasma and urine concentrations of TB-840 were determined following a validated liquid chromatography–tandem mass spectrometry (LC-MS/MS) method at the Global Clinical Central Lab Co., Ltd. (Yongin-si, Republic of Korea), Central Laboratory. The analytical system comprised an Exion LC AD liquid chromatograph coupled to a Triple Quad 5500 mass spectrometer (AB Sciex, Framingham, MA, USA). TB-840-d5 was used as an internal standard (IS) for the analysis. Sample preparation involved protein precipitation of 50 µL of plasma with acetonitrile. Chromatographic separation was performed on a Cadenza CD-C18 column (2 mm × 150 mm, 3 µm; Imtakt, Portland, OR, USA) with a gradient mobile phase consisting of 5 mM ammonium acetate in water and acetonitrile. The flow rate was maintained at 0.35 mL/min. The mass spectrometer was operated in the positive electrospray ionization (ESI) mode with multiple reaction monitoring (MRM). The precursor-to-product-ion transitions were monitored at *m*/*z* 287.0→197.0 for TB-840 and *m*/*z* 292.0→202.2 for the internal standard, TB-840-d5.

For the plasma concentration of TB-840, the calibration curve was linear over the range of 2–4000 ng/mL. The inter-run accuracy and precision evaluated at seven quality control concentrations (2, 10, 40, 200, 1000, 2000, and 4000 ng/mL) were within 98.33–101.47% and 0.57–2.91% CV, respectively. The lower limit of quantification (LLOQ) was set at 2 ng/mL.

The calibration curve for the urine concentration of TB-840 was linear over the range of 0.05–100 ng/mL. The inter-run accuracy and precision evaluated at seven quality control concentrations (0.05, 0.25, 1, 5, 25, 50, and 100 ng/mL) were within 97.29–101.67% and 0.55–4.26% CV, respectively. The LLOQ was established at 0.05 ng/mL.

### 2.4. Pharmacokinetics Analysis

PK was evaluated based on the plasma and urine concentrations of TB-840. Serial blood samples were collected in heparinized tubes before dosing and at 0.25, 0.5, 0.75, 1, 1.5, 2, 2.5, 3, 4, 6, 8, 10, 12, 24, 36, 48, 60, and 72 h after dosing. The samples were centrifuged within 30 min of blood collection at 3000 rpm for 10 min at 4 °C. The plasma was then transferred to four aliquots (1 mL each) and stored at <−70 °C. Urine samples were collected at specified intervals (0–24, 24–48, and 48–72 h post-dose). The anti-adsorbent and stabilizer were added at each collection time point. The weight of the urine was recorded at each collection interval. After thorough mixing, four aliquots from each interval (1 mL each) were prepared and stored at <−70 °C. All samples were subsequently transferred to the central laboratory for analysis.

The PK parameters were calculated via non-compartmental analysis (NCA) using Phoenix WinNonlin^®^ (Version 8.3, Certara, Princeton, NJ, USA). The following plasma PK parameters were determined: maximum observed plasma concentration (C_max_); time to reach C_max_ (T_max_); area under the plasma drug concentration–time curve from time 0 to the last quantifiable concentration (AUC_last_); area under the plasma drug concentration–time curve from time 0 to infinity (AUC_inf_); terminal elimination half-life (t_1/2_); apparent total clearance (CL/F); and apparent volume of distribution (V_d_/F). For the urinary PK parameters, the fraction of the dose excreted unchanged in urine (f_e_) and renal clearance (CL_R_) were calculated.

### 2.5. Safety and Tolerability Assessments

The safety and tolerability of the study drugs were assessed by monitoring adverse events (AEs), physical examinations, vital signs, 12-lead ECGs, and clinical laboratory tests (hematology, blood chemistry, coagulation panels, and urinalysis). All AEs were coded using the Medical Dictionary for Regulatory Activities (MedDRA, version 23.0) and evaluated by the investigator for severity and causal relationships with the study drug.

### 2.6. Statistical Analysis

Demographic characteristics and PK parameters are presented as arithmetic means with standard deviations (SDs). Baseline demographic characteristics were compared between the groups using the Kruskal–Wallis test. The dose-proportionality of TB-840 was evaluated for C_max_, AUC_last_, and AUC_inf_ by fitting a power model to the log-transformed PK parameters versus dose. The PK parameter was considered dose proportional if the 95% confidence interval (CI) for the slope of the regression line contained the value of 1. In the FE study, the effect of food was evaluated by constructing a linear mixed-effects model on the log-transformed C_max_, AUC_last_, and AUC_inf_. The geometric mean ratios (GMRs) of the fed to fasted states and their corresponding 90% CIs were calculated. A significant food effect was concluded if the 90% CI for the GMR extended beyond the prespecified 0.80–1.25 equivalence boundary. All statistical analyses were performed using SAS^®^ software (version 9.4, SAS Institute Inc., Cary, NC, USA). A *p*-value of less than 0.05 was considered statistically significant.

## 3. Results

### 3.1. Participants

**Figure 1:**
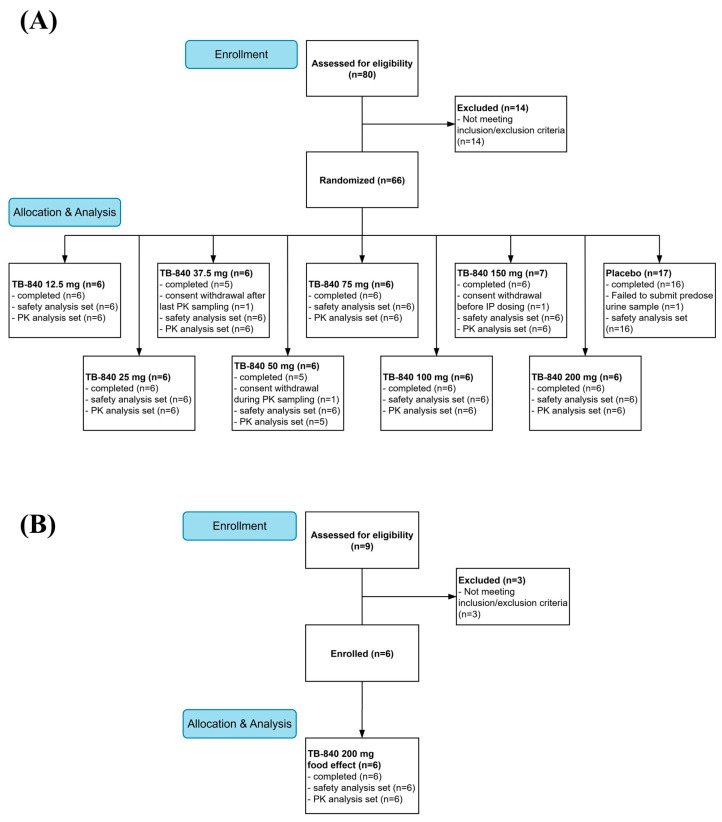
CONSORT flow diagram of study participants: (**A**) Participant disposition in the single ascending dose (SAD) part of the study. (**B**) Participant disposition in the food effect (FE) part of the study. IP, investigational product; PK, pharmacokinetic.

A total of 89 volunteers were screened for the study, of whom 72 were enrolled and randomized between 14 September 2021 and 29 August 2022. Two participants who were enrolled in the SAD study withdrew before dosing and were replaced. Following administration, two participants withdrew from SAD. All participants in the FE group completed the study. Overall, 68 of the 70 dosed participants completed all the study procedures. Consequently, 70 participants (64 in the SAD group and 6 in the FE group) received at least one dose of the study drug and were included in the safety analysis. PK analysis was performed on 53 participants (47 in the SAD group and 6 in the FE group) who received TB-840. The disposition of all participants throughout the study is detailed in the CONSORT flow diagram (Figure 1).

All the participants were male of Korean ethnicity. Their mean  ±  SD age, height, weight, and BMI in the SAD component were 26.9 ± 5.3 years, 174.7 ± 5.1 cm, 71.7 ± 8.6 kg, and 23.5 ± 2.5 kg/m^2^, respectively. The corresponding values in the FE component were 27.2 ± 3.4 years, 177.6 ± 4.7 cm, 75.3 ± 9.4 kg, and 23.8 ± 2.3 kg/m^2^, respectively. There were no statistically significant differences in baseline demographic characteristics among the dose groups in the SAD component. The baseline demographic characteristics of the participants are summarized in Table 1.

### 3.2. Pharmacokinetics

**Figure 2:**
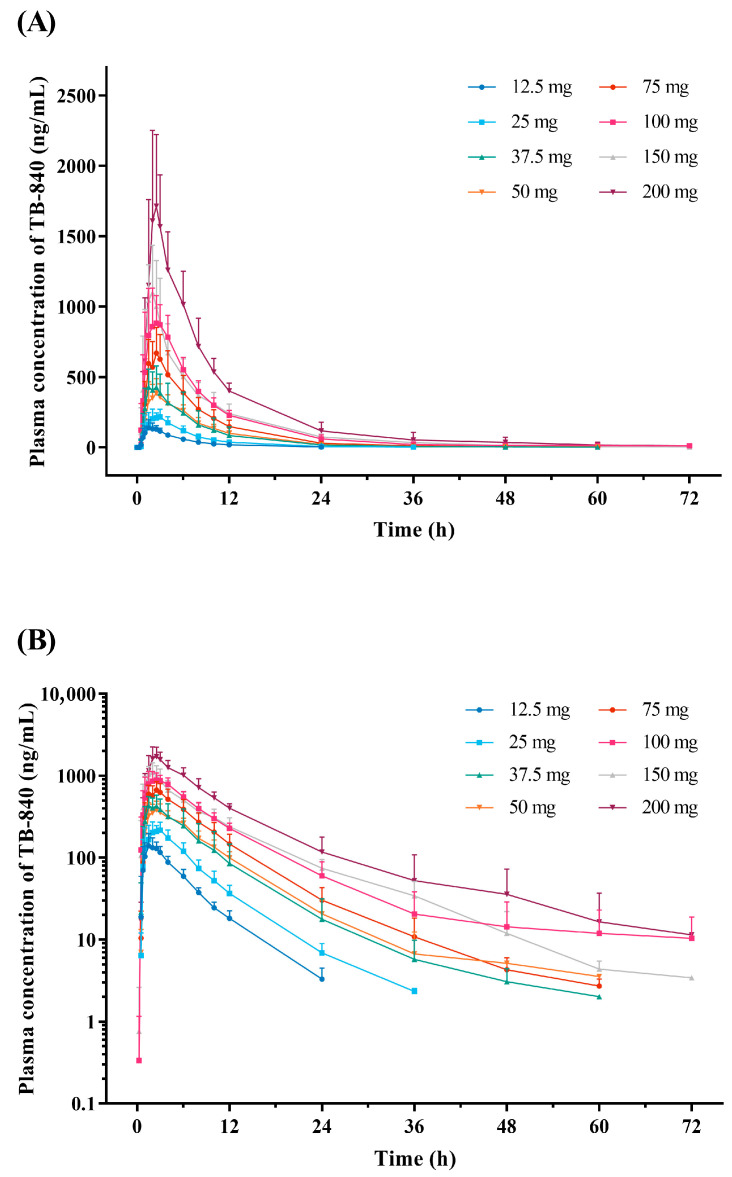
Mean plasma concentration–time profile of TB-840 following a single oral administration. Error bars denote the standard deviations: (**A**) linear scale; (**B**) semi-log scale.

Following a single oral administration, TB-840 was rapidly absorbed, with the median T_max_ ranging from 1.7 to 2.5 h across the 12.5 mg to 200 mg dose range (Figure 2). The mean terminal half-life (t_1/2_) was relatively short, ranging from 4.8 to 9.7 h. Systemic exposure increased as the dose increased from 12.5 mg to 200 mg. The mean urinary excreted fraction of TB-840 was low, indicating that renal excretion was not the major elimination pathway of TB-840. The PK parameters of the SAD study are summarized in Table 2. Dose-proportionality analysis using the power model indicated that the increase in AUC_last_ and AUC_inf_ was dose-proportional across the entire dose range, whereas the increase in C_max_ was less than proportional (Table 3).

### 3.3. Effect of Food

**Figure 3:**
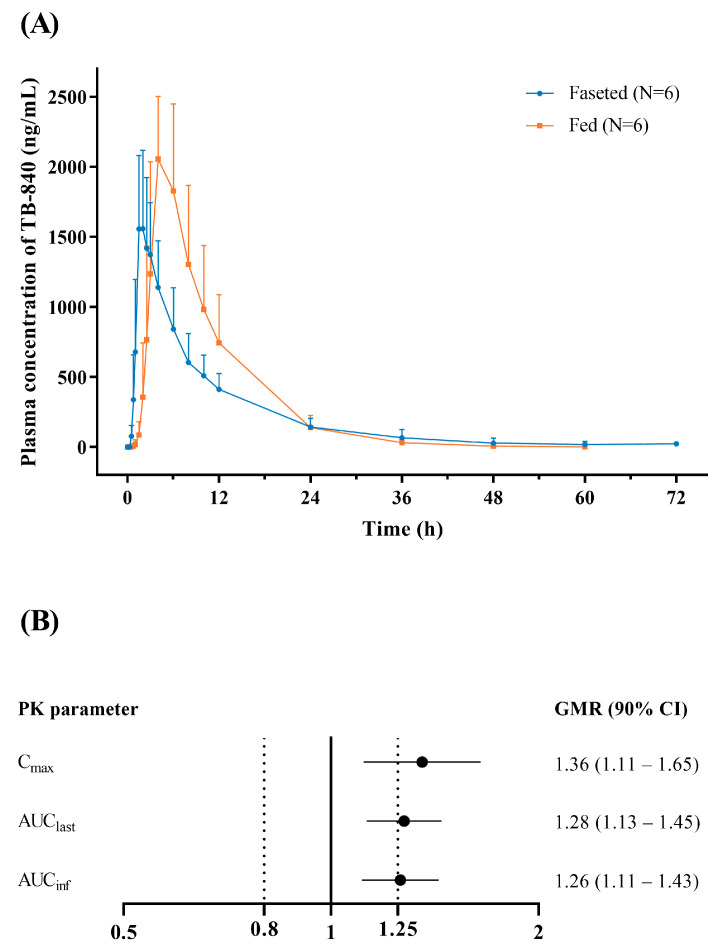
Pharmacokinetic profiles of TB-840 following oral administration with and without food. (**A**) Mean plasma concentration–time profile of TB-840 after a single 200 mg oral dose under fasted (*N* = 6) or fed (*N* = 6) state. Error bars denote the standard deviations. (**B**) Forest plot showing the geometric mean ratios (GMRs) and corresponding 90% confidence intervals (CIs) for C_max_, AUC_last_, and AUC_inf_, comparing the fed state with the fasted state. C_max_, maximum plasma concentration; AUC_last_, area under the concentration–time curve from time 0 to last time point with a measurable concentration; AUC_inf_, the AUC from time 0 to infinity.

The administration of a single 200 mg dose of TB-840 with a high-fat meal resulted in delayed absorption, increasing the median T_max_ from 1.75 h in the fasted state to 4.0 h in the fed state (Figure 3A, Table 4). Systemic exposure was also enhanced, with geometric mean ratios (fed/fasted) of 1.36 for C_max_, 1.28 for AUC_last_, and 1.26 for AUC_inf_, respectively. The 90% CIs for the three parameters fell outside the standard bioequivalence range of 0.80–1.25 (Figure 3B).

### 3.4. Safety and Tolerability

TB-840 was generally safe and well-tolerated when administered as a single oral dose of up to 200 mg. In the SAD group, four treatment-emergent adverse events (TEAEs), two cases of decreased WBC count, one case of WBC-positive urine, and one case of headache were reported in four participants across all TB-840 dose groups, with no TEAEs reported in the placebo group (Table S1). All TEAEs were transient, mild, and resolved spontaneously (Figure S1). All TEAEs were considered adverse drug reactions (ADRs). No TEAEs were reported in the FE study. No serious adverse events (SAEs) were observed. The observed laboratory abnormalities were not associated with clinical symptoms and resolved without intervention. No clinically significant changes were observed in vital signs or 12-lead ECG findings throughout this study. A review of individual laboratory data revealed isolated, transient, and asymptomatic elevations in ALT levels in three participants who received TB-840 in the SAD component. These elevations were mild, less than 1.5 times the ULN, and resolved spontaneously at the follow-up visit without any medical intervention.

## 4. Discussion

This first-in-human study evaluated the safety, tolerability, pharmacokinetics, and effect of food for TB-840, a novel RORα agonist being developed for the treatment of MASH. The principal findings indicate that TB-840 has a favorable safety and tolerability profile in healthy male subjects. Furthermore, the drug exhibited a suitable PK profile, although a significant systemic exposure effect was observed.

In this first-in-human study, single doses of TB-840 up to 200 mg were safe and well-tolerated. All four reported TEAEs were mild and transient. The most common AE, a decrease in WBC count, was considered an ADR as it could represent a pharmacodynamic effect of RORα agonism on suppression of the inflammatory response [27]. Nevertheless, the clinical significance of this decrease was considered negligible because the baseline WBC counts of the two affected participants were near the lower limit of the reference range, and the decrease was transient and resolved spontaneously. The other two TEAEs, one instance of headache and a single event of positive WBC urine, were also considered ADRs. These were likely attributable to the pleiotropic effects of RORα agonism on circadian rhythm (a known factor in migraine) and neutrophil migration, respectively [28,29]. The clinical significance of these events is limited because of their mild and self-limiting nature. Furthermore, no SAEs, dose-limiting toxicities, or discontinuation owing to adverse events were observed.

The risk of drug-induced liver injury (DILI) was prospectively monitored as an adverse event of special interest (AESI) prompted by the hepatotoxicity observed in preclinical toxicology studies of TB-840. This vigilance is particularly relevant given that other candidate therapies for MASH, such as the Farnesoid X receptor (FXR) agonist obeticholic acid, have failed to gain FDA approval, largely due to the risk of DILI [30]. The isolated, mild, and transient elevations in liver enzyme levels observed in the three participants in this study were not considered clinically significant and resolved without intervention. Although long-term administration to patients with MASH is essential for definitive safety conclusions, these initial findings demonstrate a promising safety profile that supports the continued clinical development of TB-840.

The PK profile of TB-840 was characterized by rapid absorption, with a median T_max_ of approximately 2 h and a terminal half-life ranging from 5 to 10 h. These properties are consistent with a once-daily dosing regimen, which is particularly advantageous for managing chronic diseases, such as MASH, where long-term patient adherence is crucial for therapeutic success [31]. Following single-dose administration from 12.5 mg to 200 mg, systemic exposure, as measured by the AUC, increased in a dose-proportional manner. However, substantial inter-individual variability was observed in these exposure parameters. This variability is likely attributable to individual differences in gastrointestinal absorption, first-pass metabolism, or drug-metabolizing enzyme activity such as polymorphic cytochrome P450 (CYP) enzymes, and warrants further investigation. Furthermore, the minimal renal excretion of the parent drug (<0.15%) suggests that hepatic metabolism is the primary clearance pathway, necessitating studies on potential drug–drug interactions with commonly co-administered CYP substrates, such as statins.

The administration of TB-840 with a high-fat meal resulted in a positive food effect, increasing the mean C_max_, AUC_last_, and AUC_inf_ by 36%, 28%, and 26%, respectively. However, the clinical implications of altered exposure require further investigation. Although the magnitude of the increase in exposure does not appear to preclude the administration of food, it is significant enough to be a potential source of pharmacokinetic variability. Therefore, to ensure consistent systemic exposure and minimize interindividual variability in subsequent clinical trials, TB-840 dosing should be standardized with respect to meals. The final recommendation for patients will be guided by the exposure–response relationships established in phase 2 studies for efficacy and safety.

As the first RORα agonist to be clinically evaluated for MASH, TB-840 possesses a novel mechanism of action distinct from those of other major classes of nuclear receptor modulators in development. Agents targeting PPARs, such as the pan-PPAR agonist lanifibranor, primarily improve insulin sensitivity and lipid metabolism [32]. FXR agonists such as obeticholic acid mainly regulate bile acid homeostasis and have shown antifibrotic effects [33]. In contrast, RORα activation offers a pleiotropic approach by simultaneously modulating lipid metabolism, attenuating inflammation, and regulating circadian rhythms, which are often dysregulated in MASH [16,34]. This multifaceted mechanism may offer a distinct advantage in addressing the complex pathophysiology of this disease.

Considering that MASH treatment is expected to involve combination therapies to address the complex pathophysiology of the disease, the unique mechanism of action of TB-840 makes it an attractive candidate for such regimens. For instance, its direct hepatic anti-inflammatory and metabolic effects could be synergistic with systemic metabolic agents, such as GLP-1 receptor agonists, which primarily drive weight loss and improve insulin sensitivity. A combination of TB-840 and a GLP-1 agonist could lead to greater efficacy in both MASH resolution and fibrosis regression than either agent alone. The favorable early safety and PK profile of TB-840 support its use in future combination studies.

This study had several limitations. First, the findings were obtained from healthy volunteers and may not be fully representative of the safety and pharmacokinetic profile of the target MASH population, which is typically older and presents with multiple metabolic comorbidities. Second, the study population was restricted to healthy male participants of a single ethnicity, which limits the generalizability of the results to women and other ethnic groups. Future studies utilizing more diverse populations are necessary to fully resolve these potential sources of variability. Finally, the exposure duration was limited to a single dose. Therefore, additional studies are required to investigate the long-term safety of TB-840 treatment.

The encouraging results of this Phase 1 study provide a strong foundation for the continued clinical development of TB-840. A Phase 2a study is planned for patients with biopsy-confirmed MASH to evaluate the efficacy of repeated TB-840 administration. This study will also assess the long-term safety, tolerability, and pharmacodynamic effects of noninvasive biomarkers of liver injury, inflammation, and fibrosis.

## 5. Conclusions

This first-in-human study demonstrated that the novel oral RORα agonist TB-840 was safe and well-tolerated at doses up to 200 mg. TB-840 exhibited a pharmacokinetic profile suitable for a once-daily dosing regimen. These results provide a foundation for the continued clinical development of TB-840 and support its advancement to Phase 2 trials to evaluate its efficacy and safety in patients with MASH.

## Figures and Tables

**Table 1:** Baseline demographic characteristics of study participants.

Characteristics	Single Ascending Dose (*N* = 66)	Food Effect (*N* = 6)
Age (years)	26.9 ± 5.3	27.2 ± 3.4
Male sex	66 (100)	6 (100)
Height (cm)	174.7 ± 5.1	177.6 ± 4.7
Weight (kg)	71.7 ± 8.6	75.3 ± 9.4
BMI (kg/m^2^)	23.5 ± 2.5	23.8 ± 2.3

All data are presented as means ± standard deviations, except for male sex, which is presented as n (%).

**Table 2:** Summary of pharmacokinetic parameters of TB-840 following a single oral administration.

Pharmacokinetic Parameters	12.5 mg (*N* = 6)	25 mg (*N* = 6)	37.5 mg (*N* = 6)	50 mg (*N* = 5)	75 mg (*N* = 6)	100 mg (*N* = 6)	150 mg (*N* = 6)	200 mg (*N* = 6)
C_max_ (ng/mL)	167.9 ± 37.1	268.5 ± 54.6	474.9 ± 120.1	428.0 ± 86.5	731.4 ± 155.4	985.1 ± 147.3	1172.9 ± 330.2	1803.1 ± 443.4
AUC_last_ (h∙ng/mL)	839 ± 98	1526 ± 188	3290 ± 1715	3347 ± 249	5292 ± 1231	8299 ± 1216	8782 ± 1399	14,718 ± 1800
AUC_inf_ (h∙ng/mL)	863 ± 102	1557 ± 203	3316 ± 1712	3382 ± 265	5330 ± 1244	8420 ± 1278	8823 ± 1395	14,794 ± 1815
T_max_ (h)	1.7 (0.7–2.9)	2.5 (1.5–3.0)	1.8 (1.5–2.5)	2.0 (1.5–2.5)	1.8 (1.5–2.5)	2.2 (1.5–4.0)	1.7 (1.5–2.5)	2.3 (2.0–4.0)
t_1/2_ (h)	4.8 ± 0.9	5.1 ± 0.9	5.8 ± 1.5	5.9 ± 1.8	6.7 ± 2.2	9.7 ± 7.1	7.2 ± 2.7	6.4 ± 1.6
CL/F (L/h)	14.6 ± 1.6	16.3 ± 2.0	13.4 ± 5.1	14.9 ± 1.1	14.7 ± 3.5	12.1 ± 2.0	17.4 ± 2.7	13.7 ± 1.9
V_d_/F (L)	101.8 ± 21.8	121.2 ± 28.7	111.0 ± 47.0	124.6 ± 32.3	138.8 ± 45.9	162.4 ± 106.3	184.1 ± 85.2	127.0 ± 38.9
f_e_ (%)	0.13 ± 0.04	0.13 ± 0.07	0.09 ± 0.04	0.12 ± 0.06	0.14 ± 0.07	0.1 ± 0.04	0.14 ± 0.06	0.14 ± 0.03
CL_R_ (L/h)	0.02 ± 0.01	0.02 ± 0.01	0.01 ± 0	0.02 ± 0.01	0.02 ± 0.01	0.01 ± 0.01	0.02 ± 0.01	0.02 ± 0.01
A_e_ (μg)	16.1 ± 5.6	32.2 ± 17.8	35.6 ± 16.5	62.0 ± 31.1	108.0 ± 49.8	97.7 ± 35.1	203.5 ± 96.8	289.1 ± 56.9

All data are presented as the mean ± standard deviation, except for T_max_, which is presented as the median (minimum–maximum). C_max_, maximum plasma concentration; AUC_last_, area under the concentration–time curve from time 0 to the last time point with a measurable concentration; AUC_inf_, AUC from time 0 to infinity; T_max_, time to reach the maximum plasma concentration; t_1/2_, terminal half-life; CL/F, apparent clearance; V_d_/F, apparent volume of distribution; f_e_, urinary excreted fraction; CL_R_, renal clearance; A_e_, amount of unchanged drug excreted in urine.

**Table 3:** Assessment of dose proportionality using power model.

Pharmacokinetic Parameters	Gradients	95% Confidence Intervals
C_max_	0.8367	0.7571–0.9162
AUC_last_	1.0165	0.9391–1.0939
AUC_inf_	1.0091	0.9316–1.0866

C_max_, maximum plasma concentration; AUC_last_, area under the concentration–time curve from time 0 to the last time point with a measurable concentration; AUC_inf_, AUC from time 0 to infinity.

**Table 4:** Summary of pharmacokinetic parameters of TB-840 following administration of TB-840 200 mg under fasted or fed state.

Pharmacokinetic Parameters	Fasted State (*N* = 6)	Fed State (*N* = 6)
C_max_ (ng/mL)	1741.7 ± 329.0	2353.2 ± 412.7
AUC_last_ (h∙ng/mL)	14,703 ± 3990	18,858 ± 5466
AUC_inf_ (h∙ng/mL)	14,921 ± 4105	18,891 ± 5470
T_max_ (h)	1.8 (1.5–3.0)	4.0 (3.0–6.0)
t_1/2_ (h)	9.9 ± 6.3	5.0 ± 0.5
CL/F (L/h)	14.3 ± 3.8	11.4 ± 3.2
V_d_/F (L)	205.4 ± 163.2	81.3 ± 20.3

All data are presented as mean ± standard deviation, except for T_max_, which is presented as median (minimum-maximum). C_max_, maximum plasma concentration; AUC_last_, area under the concentration–time curve from time 0 to the last time point with measurable concentration; AUC_inf_, AUC from time 0 to infinity; T_max_, time to reach the maximum plasma concentration; t_1/2_, terminal half-life; CL/F, apparent clearance; V_d_/F, apparent volume of distribution.

## Data Availability

The original contributions presented in this study are included in the article/supplementary material. Further inquiries can be directed to the corresponding authors.

## Supplementary Materials

The following supporting information can be downloaded at https://www.mdpi.com/article/10.3390/life15091410/s1. Table S1: Summary of treatment-emergent adverse events following a single oral administration of TB-840 and following administration of TB-840 at fasted or fed state. Figure S1: Timeline of Treatment-Emergent Adverse Events (TEAEs) by Dose Group. Each horizontal row represents a single participant, organized by dose group. The x-axis indicates time in days relative to the first administration of the study drug (▲). Blue and gray horizontal bars depict the duration of TEAEs and the follow-up period, respectively.
