# Supplementary material for: Safety, Pharmacokinetics, and Food Effect of the RORα Agonist TB-840, a Novel Candidate for Metabolic Dysfunction-Associated Steatohepatitis (MASH): A Randomized First-in-Human Study in Healthy Volunteers
Source: Life (Basel). 2025 Sep 7;15(9):1410. doi: 10.3390/life15091410 (PMC12471887; doi:10.3390/life15091410)
Supplement: Supplementary file 1 [file life-15-01410-s001.zip › life-3817861-supplementary.pdf]

Table S1. Summary of treatment-emergent adverse events following a single oral administration of TB-840 and following administration of TB-840 at fasted or fed state.

**Dose groups**	**Treatment-emergent adverse events**		
	**White blood cell count decreased**	**White blood cells urine positive**	**Headache**
**Single ascending dose study**			
12.5 mg (N=6)		-	1 [1]
25 mg (N=6)	-	-	-
37.5 mg (N=6)	-	-	-
50 mg (N=6)	-	1 [1]	-
75 mg (N=6)	-	-	-
100 mg (N=6)	1 [1]	-	-
150 mg (N=6)	1 [1]	-	-
200 mg (N=6)	-	-	-
Placebo (N=16)	-	-	-
**Food effect study**			
200 mg (N=6)	-	-	-

All data are presented as number of subjects [number of events].

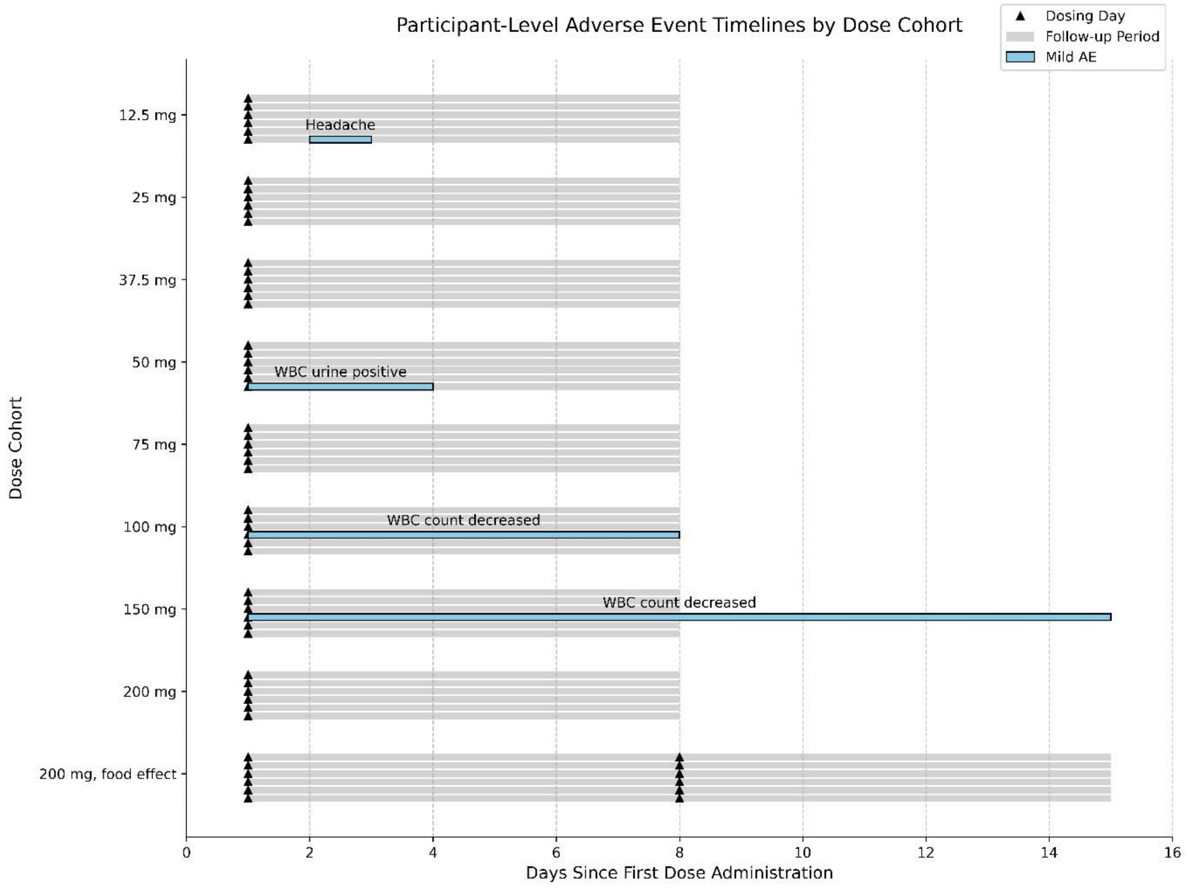

Figure S1. Timeline of Treatment-Emergent Adverse Events (TEAEs) by Dose Group. Each horizontal row represents a single participant, organized by dose group. The x-axis indicates time in days relative to the first administration of the study drug (▲). Blue and gray horizontal bars depict the duration of TEAEs and the follow-up period, respectively.
